# Physical Activity Scale for the Elderly: Translation, Cultural Adaptation, and Validation of the Italian Version

**DOI:** 10.1155/2018/8294568

**Published:** 2018-08-08

**Authors:** Antonio Covotta, Marco Gagliardi, Anna Berardi, Giuseppe Maggi, Francesco Pierelli, Roberta Mollica, Julita Sansoni, Giovanni Galeoto

**Affiliations:** ^1^Sapienza, University of Rome, Italy; ^2^Policlinico” Umberto I, Sapienza University of Rome, Italy; ^3^IRCCS Neuromed, Pozzilli, Italy; ^4^Department of Medical Surgical Sciences and Biotechnologies, Sapienza University of Rome, Italy; ^5^Department of Anatomical, Histological, Forensic and Orthopaedic Sciences, “Sapienza” University of Rome, Italy; ^6^Department of Public Health, Sapienza University of Rome, Italy

## Abstract

**Objective:**

The aim of the study was to translate and culturally adapt the Physical Activity Scale for the Elderly into Italian (PASE-I) and to evaluate its psychometric properties in the Italian older adults healthy population.

**Methods:**

For translation and cultural adaptation, the “Translation and Cultural Adaptation of Patient-Reported Outcomes Measures” guidelines have been followed. Participants included healthy individuals between 55 and 75 years old. The reliability and validity were assessed following the “Consensus-Based Standards for the Selection of Health Status Measurement Instruments” checklist. To evaluate internal consistency and test-retest reliability, Cronbach's *α* and Intraclass Correlation Coefficient (ICC) were, respectively, calculated. The Berg Balance Score (BBS) and the PASE-I were administered together, and Pearson's correlation coefficient was calculated for validity.

**Results:**

All the PASE-I items were identical or similar to the original version. The scale was administered twice within a week to 94 Italian healthy older people. The mean PASE-I score in this study was 159±77.88. Cronbach's *α* was 0.815 (p < 0.01) and ICC was 0.977 (p < 0.01). The correlation with the BBS was 0.817 (p < 0.01).

**Conclusions:**

The PASE-I showed positive results for reliability and validity. This scale will be of great use to clinicians and researchers in evaluating and managing physical activities in the Italian older adults population.

## 1. Introduction

Over the last 30 years, several studies have shown that physical activity can prevent age-related diseases, such as cardiovascular disease [1–3], diabetes mellitus [3–7], certain types of cancer [8], osteoporosis [9–11], respiratory disease [12, 13], and dementia [14, 15]. It has also been established that physical activity can improve body mass index (BMI) [16] and mental health [17] and conserve energy balance, reduce the risk of falling [18], and help a person to extend their life expectancy and maintain their independence [19].

As in other developed countries, the age ratio in present-day Italy is strongly imbalanced. In 2015, people aged 65 and older accounted for 22% of the population, and this figure is estimated to increase to 33% by 2065 [20]. The percentage of Italian older people who follow the World Health Organization's (WHO) [21] recommended levels of physical activity for adults aged 65 and older is still very low [22]. In Italy, the number of people between the ages of 65 and 74 years who claimed to take part in physical activities reached 11% in 2015, which was 60% higher than in 2005. Sports participation tends to decrease with age. From the age of 65, almost half of the population declares themselves to be sedentary (45%), and the most sedentary people are 75 years old or older (70%). However, there has been a strong increase in the older adults population's participation in sports over the last 10 years, which has almost doubled from 6% to 11% in that time [22].

As the older adults population in Italy has increased, the concepts of healthy aging and chronic disease protection have become more important. Knowledge of older adults' physical activity levels is vital to determining their health statuses and protective and preventive approaches to chronic disease. In other words, it is both necessary and important to record and measure the current physical activity levels of Italian older adults. The cross-cultural adaptation of a health status measurement tool for use in multiple countries is also essential. It is now recognized that if measurements are to be used across cultures, the items must not only be well translated but also culturally adapted so that the content validity of the instrument is maintained at a conceptual level from culture to culture.

To assess physical activity, this study used objective tools, such as accelerometers, and subjective tools that featured self-report measurements. In 2012, Williams et al. [23] conducted a systematic review and analysis of 104 self-reportable physical activity measurements, which included 35 items that had been designed to assess an older adults population. Of these, the Physical Activity Questionnaire [24], which was composed of 55 questions, and the Physical Activity Scale for the Elderly (PASE), which was composed of only 10 items, were the only scales of measurement that could be self-administered. Compared to other measurement scales, the advantages of the PASE include its brevity (five minutes), easy scoring process, and application by way of letter or phone. Furthermore, the PASE evaluates activities other than exercise. The inclusion of activities common to most older adults, such as household and caregiving activities, helps to ensure that the instrument provides a comprehensive assessment of overall physical activity.

The PASE was developed in 1993 by Washburn et al. [25], specifically to assess physical activity in epidemiologic studies of the older people. In 1999, Washburn et al. [26] evaluated the PASE to quantify the level, duration, and frequency of the physical activity that older adults engaged in. The PASE consists of 10 items that focus on the following 3 domains of activity over a period of 7 days: leisure (5 components), household (4 components), and work-related (1 component) activities. Participation in leisurely activities is recorded by frequency (e.g., never, seldom, sometimes, and often) and duration (e.g., less than an hour, 2–4 hours, or more than 4 hours); paid or unpaid work is recorded by total hours of work per week; and housework, lawn work, home repair, outdoor gardening, and care for others are recorded with yes or no answers. The total PASE score is calculated by multiplying activity participation (yes/no) or the amount of time spent on each activity (hours/week) by the weights of the items that were obtained in the original study.

The PASE was originally developed for use in Britain and North America [25, 26] and has since seen use in the Netherlands [27], Japan [28], Canada [29], China [30], Malaysia [31], and Turkey [32]. The validity and reliability of PASE were also examined in the U.S. in 1999 for patients with knee pain and physical disability [33], in 1998 for patients with moderate to severe chronic obstructive pulmonary disease [34], and in 2001 for patients with end-stage renal disease [35]. Beyond the aforementioned countries, its validity and reliability were tested in Norway in 2012 for patients with hip osteoarthritis [36], in Taiwan in 2013 for cancer survivors [37], in Switzerland in 2014 for patients who had undergone total knee arthroplasties [38] and total hip arthroplasties [39], and in Australia in 2015 for lung cancer survivors [40].

Over the past three years, several studies have used the PASE to assess the Italian population [41–48]. In spite of this, the lack of an Italian version of the scale that has been adapted to account for the country's culture has prevented its regular use in studies that assess Italy's older adults population. Thus, this study aims to translate the current PASE scale into Italian, to culturally adapt the tool, and to assess its validity and reliability.

## 2. Methods

This study was conducted by a research group composed of medical doctors and rehabilitation professionals from the “Sapienza” University of Rome and from “Rehabilitation and Outcome Measure Assessment” (ROMA) association. ROMA association in the last few years has dealt with the validation of many outcome measures in Italy [49–57].

Once the consent of the developers of the PASE is received, following the “Translation and Cultural Adaptation of Patient-Reported Outcomes Measures—Principles of Good Practice” guidelines [58] the original tool was translated from English to Italian.

*Translation and Cultural Adaptation.* The original English version of the PASE [25] was translated into three independent Italian literal translations by one Italian physiotherapist familiar with English and two native English speakers. The results were synthesized by an independent native speaker of the target language who had not been involved in the forward translations. Without having seen the original version, three Italian translators then translated the questionnaire back into English. The original version and the back-translated version of the tool were then compared. Finally to adapt the translated version to the Italian culture, a focus group composed of two physiotherapists and a proofreader familiar with both English and Italian checked the final translation and corrected any remaining spelling, diacritical, grammatical, or other errors and then reworded and reformulated some items to minimize any differences from the original English version.

*Participants.* According to preceding validations of the PASE [25–32] to be included in the study participants had to be aged 55 to 75; have their personal physician's clearance; have adequate mental status; and have no evidence of clinical depression.

Individuals with emotional or psychiatric problems (as determined by clinical screening) were excluded from the study. All participants were informed about the study, and their interest in taking part in it was recorded; those who entered the study gave their consent before inclusion [59]. Recruited participants who met the study inclusion criteria were scheduled for two testing sessions. Following the “Consensus-Based Standards for the Selection of Health Status Measurement Instruments” (COSMIN) checklist [60], the reliability and validity of the culturally adapted scale were assessed.

*Reliability.* The PASE-I was given to the population by two physiotherapists. The internal consistency of the PASE-I was examined by Cronbach's alpha (*α*) that should be at least 0.7 as an indicator of the satisfactory homogeneity of the items within the total scale [61]. The PASE-I was administered twice within a week to a representative, randomized subgroup of the population by the same professionals. To measure test-retest reliability, the Intraclass Correlation Coefficient (ICC) was calculated and the scale was considered stable with an ICC of > 0.70.

*Validity.* According to the original validation [26], concurrent validity was assessed using Pearson's correlation analyses to determine the association between the PASE-I and the Italian version of the Berg Balance Scale (BBS) [62, 63]. The BBS is a 12-item questionnaire designed to assess the subject's ability to successfully complete tasks such as standing from a sitting position, turning to look behind them, standing with their eyes closed, and standing on one foot. Each item is scored on a 0–4 metric with possible total scores ranging from 0 to 48 [62]. The Italian version of the BBS and the PASE-I were administered together by the same rater.

All statistical analyses were done using IBM-SPSS version 23.00.

## 3. Results

*Translation and Cultural Adaptation.* Following guidelines for translation and cultural adaptation,[40] after forward and backward translation, and after a consensus meeting, the translated scale was formed and all item results were identical or similar to the original version. However, the experts agreed that some of the examples used to describe leisure time activities needed an adaptation to the Italian culture to improve comprehensibility and applicability. Activities such as shuffleboard, baseball, and softball were likely to be unknown or not very common to individuals living in Italy and may not have been reflective of values related to the Italian population. Therefore, we deleted or modified examples in items 3, 4, 5, and 6 (e.g., hunting has been changed with dancing or shuffleboard has been changed with boules).

*Participants.* Participants were community-dwelling older adults recruited from September 2017 through a primary care doctor in Rome. Of the 100 recruited participants, 94 met the inclusion criteria; all agreed to participate and were enrolled in the study (mean age ± standard deviation (SD) = 62.88±7.16). The Italian version of the PASE (PASE-I) was administered from September 29, 2017. The demographic characteristics of the participants are summarized in Table 1, and the mean ± SD of the PASE-I is summarized in Table 2.

*Reliability.* The PASE-I was found to have a good degree of internal consistency, with a Cronbach's *α* of 0.815 (p < 0.01). A randomized subgroup of the population (n = 48) was involved in the test-rest reliability procedures. The PASE-I was reliable with respect to test-retest reliability with an ICC of 0.977 (p < 0.01) and > 0.967 (p < 0.01) in each domain, as reported in Table 3.

*Validity.* The Italian version of the BBS [63] was also administered to the population. The Pearson's correlation coefficient of the total score of the PASE-I with the Italian version of the BBS [63] was 0.817 (p < 0.01), indicating that the PASE-I has good concurrent validity. The Pearson's correlation coefficient of each item is reported in Table 4.

## 4. Discussion

We translated the PASE to Italian (PASE-I) and adapted it to Italian culture. The PASE is brief (five minutes) and easily scored [25]. Its brevity makes its use in large-scale epidemiologic studies, where there is limited time to assess physical activity, feasible [25]. This instrument, which has not previously been made available in Italian, is well-suited to studies on the health and physical activity of older adults populations.

*Comparison with Other Studies.* In this study, we assessed the validity and reliability of the PASE-I while working with 94 healthy and Italian older adults. The results of the study suggest that the Italian version of the tool has strong measurement properties and that it is valid and reliable for research and practice fields.

While the mean PASE scores in previous studies have varied between 94.96 [31] and 131.3 [29], the mean PASE score in this study was 159 ± 77.88. As with other studies [25–32], the current study identified a significant decline of physical activity with age. For example, adults who were 75-years old or older exhibited lower levels of physical activity than those who were 65 years old or younger. The higher total score in this study may have been linked to the participants' younger mean age (62.88 ± 7.16). The greatest contributor to the total physical activity score was household activity (52.8%), which is in line with the results from previous studies [25, 28, 31]. As in other studies [25–32], the PASE-I scores also did not significantly differ between men and women.

Lolan et al.'s [64] and Ayvat et al.'s [32] studies are the only previous studies to have calculated Cronbach's alpha (0.73 and 0.71, respectively). Regardless, the value for Cronbach's alpha in the current study was higher (0.815), which could be attributed to the rigorous cultural adaptation process that was involved in the translation of the examples that were used to describe leisurely activities.

As in other studies, we included the BBS to determine the association between balance and PASE scores. The correlation between static balance and PASE scores was discussed in the original PASE article [26] and subsequent papers. In the current study, work-related activities correlated with higher BBS scores, which is consistent with the findings from previous studies. According to existing research, individuals who more confidently perform activities that challenge their balance have also been proven to be more physically active [26, 28, 29].

*Limitations of the Study.* As in previous studies, limitations include our exclusion of individuals with mobility issues and cognitive impairment [29]. Expanding the study to include all older people with various health statuses and living conditions could help to form a database that considers a wider older adults population [32].

We agree with the authors of previous validation studies [27–32] in that the PASE itself also contains potential limitations. For example, one limitation is that leisurely activities can be influenced by climate. Furthermore, while the last item on the scale has four possible answers that gauge difficulty, this information excludes those who have worked while sitting (e.g., in an office) and is only assessed if a participant has or has not worked in the past seven days. Assessments of physical activity in the older adults could be improved if they were to differentiate between those who work standing up (answer 2) and those whose work involves carrying loads that exceed 22 kg (answer 4). The current study only compared the Italian version of the PASE with studies that evaluated balance. As such, other objective measurements should be considered to provide more precise and accurate estimates of physical activity and to reduce measurement errors that are related to these issues.

## 5. Conclusions

As the older adults population has increased, the concepts of healthy aging and chronic disease protection have become more important. To determine the health status of older adults and protective and preventive strategies against chronic disease, it is important to identify the levels of physical activity that older adults practice. As such, the present study can guide Italian physiotherapists and other health and rehabilitation professionals who work in this area [32]. To inform health policy recommendations, the use of a culturally appropriate instrument is also critical to obtaining valid population-based physical activity data. In conclusion, the Italian version of the PASE is a valid and reliable tool for the evaluation and measurement of physical activity levels in Italian older adults. Thus, this scale can prove useful to clinicians and researchers who have been charged with evaluating and managing the physical activities of the Italian older adults population.

## Figures and Tables

**Table 1:** Demographic characteristics for the 194 participants in the reliability study (PASE-I).

	Sample = 96
Age Mean (SD)	62.88 (7.16)

Gender men %	50.5

Education (%)	

Elementary school 4% Middle school 12% High school 36.4% University 47.5%	4 12 36.5 47.5

Marital Status (%)	

Married/Cohabitant Single/Widow/Divorced	88.9 11.1

Cardio circulatory Disease (%)	

Yes No	18.2 81.8

Hypertension (%)	

Yes No	36.7 63.3

Neoplasms	

Yes No	4 96

Recent Recovery (last 3 years) (%)	

Yes No	20.2 79.8

Arthritis (%)	

Yes No	5.1 94.9

Weekly Working Hours (%)	

0 1-39 More than 40	40.4 32.3 27.3

Smoker (%)	

Yes No	16.2 83.8

**Table 2:** Mean±SD PASE-I scores and BBS scores.

	Test Mean±Standard Deviation
PASE LEISURE TIME ACTIVITIES	29.94±29.63

PASE HOUSE HOLD ACTIVITIES	84.55±45.41

PASE WORK-RELATED ACTIVITIES	44.51±58.86

PASE TOTAL SCORE	159±77.88

BERG BALANCE SCALE	46.63±1.72

**Table 3:** Test-retest reliability, range of ICC parameters of each item on 48 participants (PASE-I).

	Test mean±SD	Re-Test mean±SD	ICC	IC 95%
PASE LEISURE TIME ACTIVITIES	23.18±22.22	27.71±21.7	0.993	0.988-0.996

PASE HOUSE HOLD ACTIVITIES	76.67±45	78.56±46.83	0.989	0.981-0.994

PASE WORK-RELATED ACTIVITIES	45.87±59.74	49.12±61.1	0.967	0.941-0.981

PASE TOTAL SCORE	145.72±75	150.4±76.17	0.977	0.959-0.987

**Table 4:** Gold standard analysis, Pearson's correlation between PASE-I and the Italian version of BBS.*∗*p < 0.01.

	Berg Balance Scale
PASE LEISURE TIME ACTIVITIES	0.459*∗*

PASE HOUSE HOLD ACTIVITIES	0.495*∗*

PASE WORK-RELATED ACTIVITIES	0.713*∗*

PASE TOTAL SCORE	0.817*∗*

## Data Availability

The data used to support the findings of this study are available from the corresponding author upon request.

## References

[1] Berlin J. A., Colditz G. A. (1990). A meta-analysis of physical activity in the prevention of coronary heart disease. *American Journal of Epidemiology*.

[2] Leon A. S., Connett J., Jacobs D. R., Rauramaa R. (1987). Leisuretime physical activity levels and risk of coronary heart disease and death. *The Journal of the American Medical Association*.

[3] Haapanen N., Miilunpalo S., Vuori I., Oja P., Pasanen M. (1997). Association of leisure time physical activity with the risk of coronary heart disease, hypertension and diabetes in middle-aged men and women. *International Journal of Epidemiology*.

[4] Helmrich S. P., Ragland D. R., Leung R. W., Paffenbarger R. S. (1991). Physical activity and reduced occurrence of non-insulindependent diabetes mellitus. *The New England Journal of Medicine*.

[5] Manson J. E., Rimm E. B., Stampfer M. J. (1991). Physical activity and incidence of non-insulindependent diabetes mellitus in women. *The Lancet*.

[6] Manson J. E., Nathan D. M., Krolewski A. S., Stampfer M. J., Willett W. C., Hennekens C. H. (1992). A prospective study of exercise and incidence of diabetes among US male physicians. *Journal of the American Medical Association*.

[7] Hu F. B., Sigal R. J., Rich-Edwards J. W. (1999). Walking compared with vigorous physical activity and risk of type 2 diabetes in women: A prospective study. *Journal of the American Medical Association*.

[8] Shephard R. J., Futcher R. (1997). Physical activity and cancer: how may protection be maximized?. *Critical Reviews in Oncogenesis*.

[9] Cummings S. R., Kelsey J. L., Nevitt M. C., O'Dowd K. J. (1985). Epidemiology of osteoporosis and osteoporotic fractures. *Epidemiologic Reviews*.

[10] Farmer M. E., Madans J. H., Wallace R. B., Cornoni‐Huntley J., White L. R. (1989). Anthropometric indicators and hip fracture. The NHANES I epidemiologic follow-up study. *Journal of the American Geriatrics Society*.

[11] Gregg E., Cauley J., Seeley D. (1998). Physical activity and osteoporotic fracture risk in older women. Study of Osteoporotic Fractures Research Group. *Annals of Internal Medicine*.

[12] Lacasse Y., Wong E., Guyatt G. H., King D., Cook D. J., Goldstein R. S. (1996). Meta-analysis of respiratory rehabilitation in chronic obstructive pulmonary disease. *The Lancet*.

[13] Garcia-Aymerich J., Lange P., Benet M., Schnohr P., Antó J. M. (2007). Regular physical activity modifies smoking-related lung function decline and reduces risk of chronic obstructive pulmonary disease: a populationbased cohort study. *American Journal of Respiratory and Critical Care Medicine*.

[14] US Department of Health & Human Services (1996). *Physical Activity and Health: A Report of The Surgeon General*.

[15] Reiner M., Niermann C., Jekauc D., Woll A. (2013). Long-term health benefits of physical activity: a systematic review of longitudinal studies. *BMC Public Health*.

[16] Kvamme J.-M., Wilsgaard T., Florholmen J., Jacobsen B. K. (2010). Body mass index and disease burden in elderly men and women: The Tromsø Study. *European Journal of Epidemiology*.

[17] Parker S. J., Strath S. J., Swartz A. M. (2008). Physical activity measurement in older adults: Relationships with mental health. *Journal of Aging and Physical Activity*.

[18] Ganz D. A., Alkema G. E., Wu S. (2008). It takes a village to prevent falls: Reconceptualizing fall prevention and management for older adults. *Injury Prevention*.

[19] Chad K. E., Reeder B. A., Harrison E. L. (2005). Profile of physical activity levels in community-dwelling older adults. *Medicine & Science in Sports & Exercise*.

[20] https://www.istat.it/it/anziani/popolazione-e-famiglie

[21] http://www.who.int/dietphysicalactivity/factsheet_olderadults/en/

[22] https://www.istat.it/it/files/2017/10/Pratica-sportiva2015.pdf?title=La+pratica+sportiva+in+Italia++-+19%2Fott%2F2017+-+Testo+integrale++e+nota+metodologica.pdf

[23] Williams K., Frei A., Vetsch A., Dobbels F., Puhan M. A., Rüdell K. (2012). Patient-reported physical activity questionnaires: A systematic review of content and format. *Health and Quality of Life Outcomes*.

[24] Liu B., Woo J., Tang N., Ng K., Ip R., Yu A. (2001). Assessment of total energy expenditure in a Chinese population by a physical activity questionnaire: Examination of validity. *International Journal of Food Sciences and Nutrition*.

[25] Washburn R. A., Smith K. W., Jette A. M., Janney C. A. (1993). The physical activity scale for the elderly (PASE): Development and evaluation. *Journal of Clinical Epidemiology*.

[26] Washburn R. A., McAuley E., Katula J., Mihalko S. L., Boileau R. A. (1999). The Physical Activity Scale for the Elderly (PASE): Evidence for validity. *Journal of Clinical Epidemiology*.

[27] Schult A. J., Schonten E. G., Westerterp K. R., Saris W. H. M. (1997). Validity of the Physical Activity Scale for the Elderly (PASE): According to energy expenditure assessed by the doubly labeled water method. *Journal of Clinical Epidemiology*.

[28] Hagiwara A., Ito N., Sawai K., Kazuma K. (2008). Validity and reliability of the Physical Activity Scale for the Elderly (PASE) in Japanese elderly people. *Geriatrics & Gerontology International*.

[29] Vaughan K., Miller W. C. (2012). Validity and reliability of the Chinese translation of the Physical Activity Scale for the Elderly (PASE). *Send to Disabil Rehabil*.

[30] Ngai S., Cheung R., Lam P., Chiu J., Fung E. (2012). Validation and reliability of the Physical Activity Scale for the Elderly in Chinese population. *Journal of Rehabilitation Medicine*.

[31] Ismail N., Hairi F., Choo W. Y., Hairi N. N., Peramalah D., Bulgiba A. (2015). The Physical Activity Scale for the Elderly (PASE): Validity and reliability among community-dwelling older adults in Malaysia. *Asia-Pacific Journal of Public Health*.

[32] Ayvat E., Kilinç M., Kirdi N. (2017). The Turkish version of the physical activity scale for the elderly (PASE): Its cultural adaptation, validation, and reliability. *Turkish Journal of Medical Sciences*.

[33] Martin K. A., Rejeski W. J., Miller M. E., James M. K., Ettinger W. H., Messier S. P. (1999). Validation of the PASE in older adults with knee pain and physical disability. *Medicine & Science in Sports & Exercise*.

[34] Larson J. L., Kapella M. C., Wirtz S., Covey M. K., Berry J. (1998). Reliability and Validity of the Functional Performance Inventory in Patients with Moderate to Severe Chronic Obstructive Pulmonary Disease. *Journal of Nursing Measurement*.

[35] Johansen K. L., Painter P., Kent-Braun J. A. (2001). Validation of questionnaires to estimate physical activity and functioning in end-stage renal disease. *Kidney International*.

[36] Svege I., Kolle E., Risberg M. (2012). Reliability and validity of the Physical Activity Scale for the Elderly (PASE) in patients with hip osteoarthritis. *BMC Musculoskeletal Disorders*.

[37] Su C.-C., Lee K.-D., Yeh C.-H., Kao C.-C., Lin C.-C. (2014). Measurement of physical activity in cancer survivors: A validity study. *Journal of Cancer Survivorship*.

[38] Bolszak S., Casartelli N. C., Impellizzeri F. M., Maffiuletti N. A. (2014). Validity and reproducibility of the Physical Activity Scale for the Elderly (PASE) questionnaire for the measurement of the physical activity level in patients after total knee arthroplasty. *BMC Musculoskeletal Disorders*.

[39] Casartelli N. C., Bolszak S., Impellizzeri F. M., Maffiuletti N. A. (2015). Reproducibility and validity of the physical activity scale for the elderly (PASE) questionnaire in patients after total hip arthroplasty. *Physical Therapy in Sport*.

[40] Granger C. L., Parry S. M., Denehy L. (2015). The self-reported Physical Activity Scale for the Elderly (PASE) is a valid and clinically applicable measure in lung cancer. *Supportive Care in Cancer*.

[41] Curcio F., Liguori I., Cellulare M. (2018). PASE (Physical Activity Scale for the Elderly) Score Is Related to Sarcopenia in Noninstitutionalized Older Adults. *Journal of Geriatric Physical Therapy*.

[42] Gagliardi C., Papa R., Postacchini D., Giuli C. (2016). Association between cognitive status and physical activity: Study profile on baseline survey of the my mind project. *International Journal of Environmental Research and Public Health*.

[43] Muscari A., Bianchi G., Conte C. (2015). No Direct Survival Effect of Light to Moderate Alcohol Drinking in Community-Dwelling Older Adults. *Journal of the American Geriatrics Society*.

[44] De Nunzio C., Presicce F., Lombardo R. (2016). Physical activity as a risk factor for prostate cancer diagnosis: A prospective biopsy cohort analysis. *BJU International*.

[45] Bacchi E., Bonin C., Zanolin M. E. (2016). Physical activity patterns in normal-weight and overweight/obese pregnant women. *PLoS ONE*.

[46] Tarantino U., Baldi J., Scimeca M. (2016). The role of sarcopenia with and without fracture. *Injury*.

[47] Noale M., Maggi S., Artibani W. (2017). Pros-IT CNR: an Italian prostate cancer monitoring project. *Aging Clinical and Experimental Research*.

[48] Giuli C., Papa R., Bevilacqua R. (2014). Correlates of perceived health related quality of life in obese, overweight and normal weight older adults: An observational study. *BMC Public Health*.

[49] Galeoto G., Lauta A., Palumbo A., Castiglia S. F., Mollica R., Santilli V. (2015). The Barthel Index: Italian translation, adaptation and validation. *International Journal of Neurology and Neurotherapy*.

[50] Galeoto G., Berardi A., De Santis R. (2018). Validation and cross-cultural adaptation of the Van Lieshout test in an Italian population with cervical spinal cord injury: a psychometric study. *Spinal Cord Series and Cases*.

[51] Culicchia G., Nobilia M., Asturi M. (2016). Cross-Cultural Adaptation and Validation of the Jebsen-Taylor Hand Function Test in an Italian Population. *Rehabilitation Research and Practice*.

[52] Castiglia S. (2017). The culturally adapted Italian version of the Barthel Index (IcaBI): assessment of structural validity, inter-rater reliability and responsiveness to clinically relevant improvements in patients admitted to inpatient rehabilitation centers. *Functional Neurology*.

[53] Marquez M. A., De Santis R., Ammendola V. (2018). Cross-cultural adaptation and validation of the “Spinal Cord Injury-Falls Concern Scale” in the Italian population. *Spinal Cord*.

[54] Galeoto G., Sansoni J., Scuccimarri M. (2018). A Psychometric Properties Evaluation of the Italian Version of the Geriatric Depression Scale. *Depression research and treatment*.

[55] Murgia M., Bernetti A., Delicata M., Massetti C., Achilli E. M., Mangone M. (2018). Inter-and intra-interviewer reliability of Italian version of Pediatric Evaluation of Disability Inventory. *Annali di Igiene*.

[56] Berardi A., De Santis R., Tofani M. (2017). The Wheelchair Use Confidence Scale: Italian translation, adaptation, and validation of the short form. *Disability and Rehabilitation: Assistive Technology*.

[57] Tofani M., Candeloro C., Sabbadini M. (2018). The psychosocial impact of assistive device scale: Italian validation in a cohort of nonambulant people with neuromotor disorders. *Assistive Technology*.

[58] Wild D., Grove A., Martin M. (2005). Principles of good practice for the translation and cultural adaptation process for patient-reported outcomes (PRO) measures: report of the ISPOR Task Force for Translation and Cultural Adaptation. *Value in Health*.

[59] Galeoto G., Mollica R., Astorino O., Cecchi R. (2015). Informed consent in physiotherapy: proposal of a form. *Giornale Italiano di Medicina del Lavoro ed Ergonomia*.

[60] Mokkink L. B., Terwee C. B., Patrick D. L. (2010). The COSMIN checklist for assessing the methodological quality of studies on measurement properties of health status measurement instruments: an international Delphi study. *Quality of Life Research*.

[61] Nunnally J. C. (1978). *Psychometric Theory*.

[62] Berg K. O., Wood-Dauphinee S. L., Williams J. I., Maki B. (1992). Measuring balance in the elderly: Validation of an instrument. *Canadian Journal of Public Health*.

[63] Ottonello M., Ferriero G., Benevolo E., Sessarego P., Dughi D. (2003). Psychometric evaluation of the Italian version of the Berg Balance Scale in rehabilitation inpatients. *European Journal of Physical and Rehabilitation Medicine*.

[64] Loland N. W. (2002). Reliability of the Physical Activity Scale for Elderly (PASE). *European Journal of Sport Science*.

